# Role of Probiotics in Enhancing Immune Function and Improving the Effectiveness of Treatments for Pancreatic Cancer

**DOI:** 10.3390/microorganisms13122687

**Published:** 2025-11-25

**Authors:** Kawaljit Kaur

**Affiliations:** ImmuneLink, LLC, Riverside, CA 92508, USA; drkawalmann@g.ucla.edu; Tel.: +1-5093395967

**Keywords:** pancreatic cancer, NK cells, probiotics, immunotherapy, immune cells, cytotoxicity, IFN-γ

## Abstract

Pancreatic cancer often goes unnoticed in its early stages because it causes few or no symptoms, leading to late diagnoses and limited treatment options. The main challenges are delayed detection, drug resistance, and the tumor’s complexity, though progress is being made in targeted therapies, immunotherapy, metabolism-based strategies, and early detection methods. Current treatments aim to boost immune responses, extend survival, and improve quality of life. In pancreatic cancer patients, peripheral blood-derived natural killer (NK) cells show reduced numbers, decreased cytotoxic activity, and lower cytokine secretion, which may contribute to tumor growth and spread. NK cell-based immunotherapies have gained attention, with in vitro and mouse studies showing that NK cells can slow the growth of pancreatic tumor stem-like cells and encourage tumor differentiation through cytokines. Preclinical research in humanized mice suggests that NK cell therapies could reduce tumor load and restore immune function. Probiotics are also being studied in preclinical models as a potential adjuvant in therapy to restore immunity, slow tumor growth, and improve outcomes. This review compiles preclinical evidence on the benefits of combining probiotics with NK cell-based treatments for pancreatic cancer. In vitro studies indicate that probiotics can activate immune cells like peripheral blood mononuclear cells (PBMCs), NK cells, T cells, and antigen-presenting cells to help fight tumors. In humanized mouse models, combining probiotics with NK cell therapy has shown promise in reducing tumor burden, restoring immune function, and even reversing tumor-induced bone damage. The exact probiotic formulations and mechanisms are still under study, and clinical trials are in early stages without conclusive results yet.

## 1. Introduction and Background

Pancreatic cancer is an aggressive disease often diagnosed late due to its vague early symptoms, diverse tumor types, and poor prognosis, with a 5-year survival rate of less than 15% [1,2]. It is one of the top causes of cancer-related deaths worldwide, with little improvement in survival rates over the years [3]. Pancreatic cancer patients often face severe nutritional deficiencies, muscle wasting, and bone issues, with the first case of bone metastasis documented in Russian literature in 1963 [4]. Common symptoms include fatigue, weakness, unexplained weight loss, abdominal pain radiating to the back, anorexia, jaundice, and digestive problems like nausea or bloating [5]. Early detection is tough because symptoms usually arise only after the tumor grows significantly or spreads. Most pancreatic tumors (90–95%) are exocrine, primarily pancreatic adenocarcinomas (PDAC), which arise from enzyme-producing cells near the ducts, grow rapidly, and spread early [6]. The remaining 5–10% are pancreatic neuroendocrine tumors (pNET), originating from hormone-producing cells, growing more slowly, and offering a better prognosis [7]. PDAC progresses due to a mix of genetic mutations, disrupted signaling pathways, tumor microenvironment interactions, and cellular adaptability [6]. The causes are not fully understood but are linked to genetic mutations, hereditary factors, smoking, obesity, heavy alcohol use, chronic pancreatitis, aging (65–74 years), family history, chemical exposure, and diabetes [8,9]. Key genetic drivers include KRAS mutations, found in about 95% of PDAC cases, which activate growth and survival pathways, along with mutations in tumor suppressor genes like TP53, CDKN2A (p16), and SMAD4, causing uncontrolled cell growth, avoidance of cell death, and tumor development [10,11]. Early detection significantly improves outcomes, and PNETs generally have better survival rates than adenocarcinomas [11].

Pancreatic cancer often spreads to the liver, peritoneal cavity, lungs, bones, adrenal glands, and brain [4,12,13,14,15,16]. The liver (50–70%) is the primary site for metastasis due to portal circulation, worsening the prognosis [12]. The peritoneum (around 50% in advanced cases) often develops carcinomatosis, causing symptoms and limiting treatment options [13]. Pulmonary metastases (15–30%) affect survival as tumor cells become trapped in the lung’s capillaries. Bone metastases (10–20%) are less common but cause pain and fractures [4]. Adrenal gland involvement (5–10%) is rare but can disrupt hormonal balance [14]. Brain metastases, occurring in less than 1% of cases, are a rare complication with poor outcomes [15,16]. Diagnosis usually involves imaging tests like computed tomography (CT) scans, magnetic resonance imaging (MRI), positron emission tomography (PET) scans, and endoscopic ultrasound. Blood tests for markers like cancer antigen 19-9 (CA 19-9) can help monitor the disease, but are not definitive, and biopsies are often guided by imaging [2].

The pancreatic tumor microenvironment (TME) is characterized by a dense fibrotic stroma, cancer-associated fibroblasts (CAFs), immune-suppressive cells like regulatory T cells and myeloid-derived suppressor cells, extracellular matrix (ECM), and cytokines [17,18,19]. This environment facilitates tumor growth, hinders drug delivery, enables immune evasion, and promotes metastasis [20]. Secreted factors and cytokines, such as TGF-β, further modify the microenvironment to foster tumor growth, fibrosis, angiogenesis, metastasis, and immune suppression [11,21]. Treatment for pancreatic cancer depends on the stage, location, and the patient’s overall health [22]. Surgery, including the Whipple procedure, distal pancreatectomy, or total pancreatectomy, offers the only potential cure but is limited to localized cases [22,23]. Chemotherapy (e.g., FOLFIRINOX, gemcitabine) and radiation therapy are used to control the disease and shrink tumors [22]. Other strategies involve MAPK pathway inhibitors like Trametinib and Binimetinib, KRAS-specific inhibitors such as Sotorasib and Adagrasib, and immunotherapy, though immune evasion remains a challenge [22,24]. Targeted therapies and immunotherapies hold promise for specific genetic subtypes or advanced cases, while palliative care focuses on relieving symptoms, particularly pain [22]. Strategies to modify the TME, using a combination of CAR-T with immune checkpoint inhibitors and cytokine modulations, are also under investigation to boost pancreatic cancer therapeutics [25].

A diet rich in probiotics has been linked to reduced rates of pancreatitis and pancreatic cancer [26,27]. Probiotics like *Lactobacillus* and *Bifidobacterium* spp. help maintain gut microbial balance, strengthen intestinal barriers, and decrease pro-inflammatory microbial products reaching the pancreas [28,29]. Animal studies indicate that probiotics may reduce pancreatic inflammation, suppress enzyme activity, and enhance proteins like occludin and claudins to prevent tumors [30]. Studies emphasized the probiotic-induced gut microbiome’s impact on tumor growth and therapy response [31], particularly in how *Lactobacillus* species activate aryl hydrocarbon receptors (AhR) in tumor-associated macrophages, leading to immunosuppression and tumor progression [32]. Probiotics, discovered by Elie Metchnikoff in the early 20th century, are crucial for maintaining gut balance [33,34]. They may influence pancreatic cancer by impacting gut microbiota, immune responses, and tumor pathways, though the evidence is mixed and context-dependent, with risks like potentially promoting tumor growth [35,36,37]. In vivo studies show that probiotic treatments can enhance tumor immune cell recruitment, boost anti-cancer activity, improve PBMC cytotoxicity and cytokine secretion, and reduce tumor burden [38,39]. Translational research is currently investigating fecal microbiota transplants and microbiome-targeted therapies to influence immune responses in pancreatic cancer patients. Early clinical trials reveal better immune markers and therapy outcomes in cancer patients using probiotics [35]. Some probiotic regimens, combined with standard therapy, have been shown to improve survival, reduce complications, and enhance quality of life [35]. Host factors like baseline microbiome, genetics, diet, and immune status also play a significant role in these effects [35]. Recent studies suggest potential benefits of probiotics in pancreatic cancer for both mouse models and humans, especially when combined with surgery, chemotherapy, radiotherapy, or natural killer (NK) cell-based therapies [38,40,41,42,43].

NK cells play a key role in inhibiting pancreatic cancer through their effector functions, such as direct cytotoxicity and antibody-dependent cellular cytotoxicity (ADCC). They also regulate or activate the anti-cancer activity of other immune effectors through the cytokines and chemokines they secrete [44,45,46]. Maintaining balanced numbers and anti-cancer activity of peripheral blood-derived NK cells, along with increased NK cell infiltration in tumor tissues, significantly enhances patient outcomes [40,47,48,49]. However, studies have shown reduced numbers and diminished anti-cancer activity of NK cells in cancer patients, both in tumor tissues and peripheral blood-derived immune subsets [47,48,49,50]. To address this, several technologies have been developed to expand and activate NK cells, enabling large-scale production for therapeutic use [50,51,52,53,54,55]. Combining probiotics with feeder cells promotes significant NK cell expansion and enhances their anti-cancer functionality [39,50,56,57].

Ongoing clinical trials suggest that probiotics might offer modest survival benefits, especially when combined with curative surgery or chemotherapy. However, their effects can vary greatly depending on the context, as some probiotic strains could potentially promote tumor growth or weaken the immune system. Using probiotics for pancreatic cancer is still experimental, and further research is needed to determine their safety, effectiveness, and the most beneficial strains. This review highlights preclinical studies showing how probiotics can activate immune cells to directly or indirectly help break down and inhibit pancreatic cancer growth. In humanized mice with pancreatic tumors, combining probiotics with NK cell therapies has shown added benefits in reducing tumor size, restoring immune function, and preventing bone loss. These findings lay the groundwork for clinical studies aimed at helping patients benefit from probiotics.

## 2. Probiotics May Help Fight Pancreatic Cancer, Either Directly or Indirectly: Insights from Preclinical Studies

Preclinical studies have shown that probiotics help balance the gut microbiome, activate peripheral blood mononuclear cells (PBMCs), reduce pathogen-induced inflammation, and lower exposure to systemic carcinogens [58,59,60]. They support the intestinal barrier and microbial balance by boosting proteins and mucus that block pathogens and prevent excessive immune reactions [58]. Through direct interaction and signaling with immune cells, probiotics enhance immunity, strengthen anti-cancer properties, and maintain gut tolerance [61,62]. Therefore, probiotics either directly combat pancreatic tumors or boost the function of the immune system against pancreatic cancer. Their benefits are strain-specific, as outlined in Table 1, with beneficial formulations including *Lactobacillus* and *Bifidobacterium* [61,63].

### 2.1. Probiotics Directly Fight Pancreatic Cancer

Probiotics may also inhibit angiogenesis, epithelial-to-mesenchymal transition (EMT), and metastasis, potentially slowing tumor growth. Using *Lactobacillus plantarum* to ferment stevia extract generated bioactive metabolite clonogenic acid methyl ester (CAME) was found to arrest pancreatic cancer cells (PANC-1) in the G0/G1 phase and induce apoptosis, suggesting its role as a pancreatic cancer therapeutic [64]. Short-chain fatty acids (SCFAs) derived from probiotics, like butyrate, acetate, and propionate, can activate tumor suppressor genes epigenetically and inhibit pancreatic cancer cell invasion and metastasis by reducing integrin β4 expression or inhibiting histone deacetylase (HDAC) activity [27,65,66]. Probiotic treatments also decrease VEGF expression in tumors, limiting angiogenesis, promoting differentiation, and inducing tumor-antigen surface expression in pancreatic tumors [67]. Research with in vitro and xenograft mouse models has shown that *Lactobacillus casei* and *Lactobacillus reuteri* may help ease pancreatic cancer by blocking TLR4, promoting macrophage M1 polarization, and supporting a healthy gut microbiome [60]. Probiotics may also reduce cancer cell growth, prevent PanIN progression, and curb metastasis through mechanisms possibly involving the TGF-β signaling pathway [68].

**Table 1:** Probiotic strains that may help inhibit pancreatic cancer and improve its prognosis.

Probiotic Strains	Mechanism of Action	References
Direct inhibition of pancreatic tumor		
*Bifidobacterium longum* *Lactobacillus lactis* *Lactobacillus reutri* *Lactobacillus plantarum* *Lactobacillus paracasei/casei*	-Inhibit pancreatic tumor proliferation	[59,60,68,69,70,71]
*Lactobacillus paracasei/casei* *Lactobacillus rhamnosus*	-Inhibit tumor growth by decreasing matrix metalloproteinase-9 (MMP-9) activity	[28,59,60,72,73,74,75,76,77]
*Lactobacillus acidophilus* *Lactobacillus plantarum* *Lactobacillus rhamnosus*	-Inactivated the NF-kB inflammatory pathway	[59,74,75,76,77,78,79,80,81]
*Lactobacillus reutri* *Lactobacillus paracasei/casei*	-Inhibit p53-p21-Cyclin B1/Cdk1 signaling pathway resulting in growth arrest at G_2_ growth phase of tumors (Ferrichrome-mediated apoptosis)	[60]
*Lactobacillus paracasei/casei*	-Activated c-jun N-terminal kinase (JNK)-mediated apoptosis of tumors	[28,59,60,72,73]
*Lactobacillus plantarum* *Aspergillus oryzae*	-P38 MAPK-mediated apoptosis	[64,82]
*Bifidobacterium longum* *Lactobacillus paracasei/casei*	-Increase efficacy of PD-1 therapy in pancreatic cancer	[28,59,60,68,69,70,71,72,73]
Indirect inhibition of pancreatic tumor		
*Clostridium butyricum* *Enterococcus faecalis* *Bacillus mesentericus*	-Increase surface expression of CD11b, HLA-DR, CD4, CD45Ram CD25, CD44 and CD69 in PBMCs	[83]
*Bifidobacterium longum* *Bifidobacterium breve* *Bifidobacterium infantis* *Lactobacillus acidophilus* *Lactobacillus lactis* *Lactobacillus plantarum* *Lactobacillus paracasei/casei* *Lactobacillus bulgaricus* *Lactobacillus rhamnosus*	-Regulate cytokine secretion in PBMCs and NK cells	[59,74,75,76,77]
*Streptococcus thermophilus* *Enterococcus faecalis* *Clostridium butyricum* *Bacillus mesentericus* *Lactobacillus plantarum* *Lactobacillus rhamnosus* *Enterococcus hirae*	-Promote Th1-type cytokine profile, increasing IL-12 and IFN-γ in PBMCs, NK and T cells	[59,74,75,76,77,83,84,85,86,87]
*Bifidobacterium infantis* *Bifidobacterium breve*	-Support Th2 profile with higher IL-10 and IL-6 compared to IL-12 and IFN-γ	[59,74,75,76,77,88,89]
*Lactobacillus lactis* *Lactobacillus rhamnosus* *Enterococcus hirae*	-Enhance Th17 immune response against cancer	[86,87]
*Streptococcus thermophilus* *Bifidobacterium longum* *Bifidobacterium breve* *Lactobacillus paracasei/casei* *Lactobacillus rhamnosus*	-Boost cytotoxic activity in PBMCs, NK and T cells	[59,84,85]
*Bifidobacterium longum* *Lactobacillus paracasei/casei* *Enterococcus hirae* *Bifidobacterium longum*	-Increase the number of total T cells, NK cells, and increase the CD8+/CD4+ T ratio	[59,69,70,71,86,87]
*Bifidobacterium breve* *Lactobacillus acidophilus* *Lactobacillus rhamnosus*	-Encourage CD4+ and CD8+ T cell proliferation	[59,90,91]
*Lactobacillus plantarum* *Lactobacillus paracasei/casei* *Bifidobacterium longum*	-Increase tumor infiltration of CD4+ and CD8+ T cells	[59,64,92,93]
*Lactobacillus casei * *Lactobacillus reuteri *	-Supression of TLR4 to promote macrophage M1 polarization	[60]
*Bifidobacterium longum* *Lactobacillus lactis* *Bacillus mesentericus*	-Increase anti-cancer gene expressions on dendritic cells	[59,69,70,71,83,94]
Boost efficacy or mimic the toxicity of other therapeutics		
*Bifidobacterium longum* *Lactobacillus acidophilus*	-Protection against chemo- and radiotherapy-induced fever and diarrhea in pancreatic cancer patients	[59,69,70,71]
*Lactobacillus acidophilus* *Lactobacillus paracasei/casei*	-Sensitized tumor cells to chemotherapy by faster activation of caspase-3 and downregulation of p21 protein	[59,78,79,80,81]
*Lactobacillus casei * *Lactobacillus reuteri *	-Regulate gut microbial homeostasis	[60]

### 2.2. Probiotics Indirectly Fight Pancreatic Cancer

**Figure 1:**
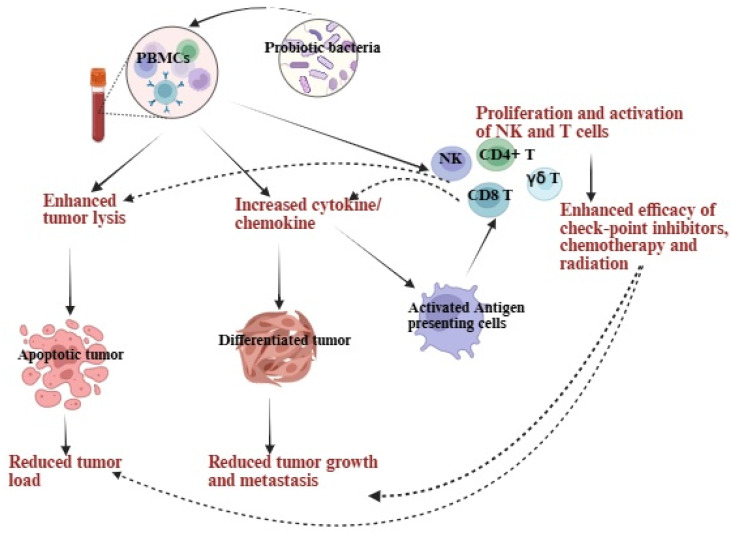
Probiotic treatments enhance cytotoxic activity, increase anti-inflammatory cytokines, decrease pro-inflammatory cytokines in PBMCs and PBMC-derived immune cells, ultimately leading to lysis and growth inhibition of pancreatic cancer. They boost the anti-cancer effects of PBMCs, as well as PBMC-derived NK and T cells, either directly or indirectly by modulating dendritic cells or other antigen-presenting cells. The heightened cytotoxic activity of PBMCs, NK, and T cells supports tumor apoptosis, while increased cytokine secretion aids in tumor differentiation. Upon differentiation, tumors proliferate and metastasize at a minimal rate. Antigen-presenting cells drive T-cell polarization and enhance T-cell cytotoxicity, working in synergy with radiotherapy, chemotherapy, and immune checkpoint inhibitors. These processes highlight the potential of probiotics to encourage apoptosis, cell cycle arrest, and tumor growth inhibition. Illustration created with https://BioRender.com on 24 September 2025. https://app.biorender.com/illustrations/68cdb701d629a499caac78aa?slideId=9ad3a503-198a-4fc5-8316-c2e0f35b40ec (accessed on 26 September 2025).

PBMCs or PBMCs-derived lymphocytes, such as T cells and NK cells, are vital for anti-tumor immunity [95,96]. Probiotics, often *Lactobacillus* and *Bifidobacterium*, interact directly or indirectly with PBMCs to enhance their anti-tumor effects [83] (Figure 1). These bacteria produce short-chain fatty acids (SCFAs) like butyrate, acetate, and propionate, which promote PBMCs or PBMCs-derived NK cells and T cells-mediated apoptosis of pancreatic cancer via Fas/FasL pathways and support T-cell differentiation and activation [97,98,99]. Probiotics also create bioactive compounds like bacteriocins and exopolysaccharides (EPS) that boost PBMC activation and selectively target tumor cells [100]. Enhanced PBMC cytotoxicity can halt tumor cell cycles at G0/G1 or G2/M phases, inhibiting tumor growth and metastasis [40]. In-vitro treatment of probiotics in PBMCs shows increased CD8+ T cell/Treg cell ratios, higher IFN-γ production by NK, CD4+ T, and γδ T cells, improved tumor recognition, and enhanced cytotoxicity and tumor growth inhibition via differentiation. Probiotic treatments increase anti-angiogenic cytokines, particularly IFN-γ and TNF-α in PBMCs, NK cells, and T cells (CD8+, CD4+, and γδ T cells); these cytokines can inhibit tumor growth by promoting differentiation [59]. Differentiated tumors proliferate more slowly and express more surface molecules like major histocompatibility complex-class I (MHC-I), CD54, and PD-L1, improving immune recognition and reducing tumor-supportive microenvironments [59,101].

Probiotics, especially lactic acid bacteria like *Lactobacillus* and *Bifidobacterium*, provide significant immunomodulatory benefits by enhancing NK cell-driven anti-cancer responses [59]. In-vitro treatment of probiotics in NK cells resulted in increased cytolytic activity of NK cells against pancreatic cancer cells, producing more perforins and granzyme B for precise cancer cell targeting [59]. They activate NK cells through cytokine stimulation, promoting tumor differentiation, growth inhibition, and improved immune recognition via upregulated expression of MHC-class I, CD54, and PD-L1 [59]. Strains like *Lactobacillus paracasei* support Th1- and Th17-mediated anti-tumor immunity, indirectly activating NK cells [59]. Probiotics also stimulate dendritic cells (DCs) and other antigen-presenting cells to present antigens more effectively, bolstering NK cells and T cells’ anti-cancer functions [95]. They enhance DC maturation by upregulating CD80 and CD86, improving antigen presentation to naive T cells. Activated DCs release cytokines like IL-10, IL-12, and TGF-β, guiding naive T cells to differentiate into regulatory T cells (Tregs) to suppress excessive inflammation [96,97]. Effector T cells, including Th1 and Th17, play a role in anti-tumor immunity and work in synergy with chemotherapy drugs like cyclophosphamide and gemcitabine [98]. These effects may boost cancer therapy by restoring immune surveillance, suppressing tumor progression, and improving outcomes [99,100,101] (Figure 1).

## 3. Probiotics, When Combined with Feeder Cells, Contribute to Improving the Development of NK Cell-Based Immunotherapies

Natural killer (NK) cells, known for their strong anti-tumor activity, can effectively target cells that lack or have altered major histocompatibility complex (MHC) class I molecules [102,103]. While NK cell-based therapies are considered safe, their effectiveness is limited due to challenges in enhancing their expansion and anti-tumor capabilities [104,105]. Autologous NK cells from pancreatic cancer patients often face impairments caused by prior immunosuppression, making their expansion more difficult compared to those from healthy donors [106,107,108]. Studies show that NK cells expanded from pancreatic cancer patients demonstrate lower cytotoxicity and reduced IFN-γ secretion compared to healthy donors [109]. These cells also exhibit decreased levels of activating receptors like CD16, CD56, Nkp30, Nkp44, Nkp46, NKG2D, and CD54 [109]. To address these issues, healthy allogeneic NK cells are being explored for cancer therapies and are under clinical investigation [110]. These allogeneic cells, sourced from peripheral blood, cord blood, hematopoietic stem cells, or induced pluripotent stem cells, can be expanded and cryopreserved for convenient use [111,112,113]. Several methods have been developed to expand NK cells ex vivo, often using feeder cells with or without cytokines and other activation signals [50,51,52,53,54,114,115,116,117,118]. Feeder cells play a vital role in activating and proliferating NK cells by providing receptor-ligand interactions and cytokine support, enabling large-scale therapeutic applications [50,119,120,121]. Cytokines like IL-2, IL-15, and IL-21, along with membrane-bound cytokines and antibodies, are commonly used to supplement feeder cell cultures [122,123]. When there’s a donor-recipient mismatch, donor NK cells with inhibitory receptors like KIR do not recognize HLA class I on recipient cells. This lack of recognition activates the donor NK cells, allowing them to attack cancer cells that lack the MHC ligands needed for KIR inhibition. As a result, alloreactive NK cells destroy these cancer cells [110,124,125]. Engineered or feeder-expanded NK cells, with enhanced KIR expression, increased cytokine secretion, improved ADCC through elevated levels of CD16 and NKG2D, reduced inhibitory receptors like NKG2A, and the ability to kill tumor cells regardless of MHC-class I expression, show great promise as allogeneic cancer therapies [50,126,127,128,129]. Additionally, the absence of graft-versus-host disease (GVHD) in NK cells ensures their safety in adoptive cell-based therapies [130,131].

**Figure 2:**
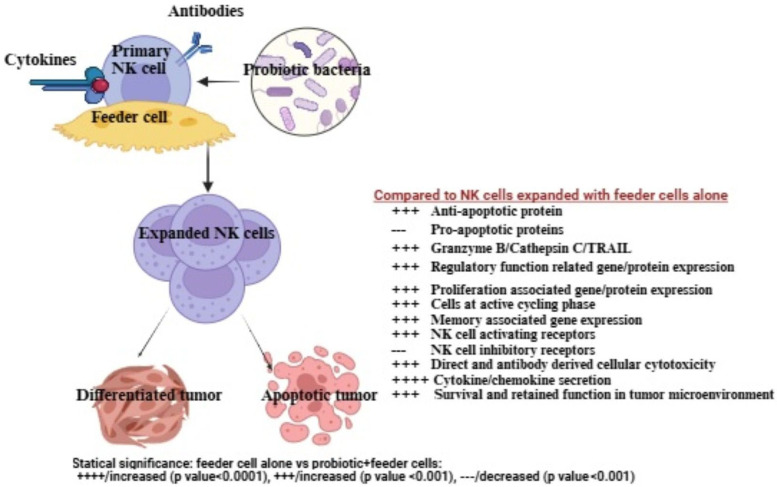
A schematic overview of the generation and characteristics of expanded NK cells using probiotics and feeder cells. Human peripheral blood-derived cells are treated with cytokines and antibodies and co-cultured with feeder cells alongside probiotic bacteria. NK cells expanded with probiotics and feeder cells showed enhanced expansion and functional activation compared to those expanded with feeder cells alone. Illustration created with https://BioRender.com on 26 September 2025. https://app.biorender.com/illustrations/68cdea1c0412ec45e81a342d?slideId=9ad3a503-198a-4fc5-8316-c2e0f35b40ec (accessed on 26 September 2025).

Combining probiotics with feeder cells has been shown to enhance the effectiveness of NK cell therapy compared to using feeder cells alone (Figure 2) [50]. Selected probiotic strains like *Streptococcus thermophilus*, *Bifidobacterium longum*, *Bifidobacterium breve*, *Bifidobacterium infantis*, *Lactobacillus acidophilus*, *Lactobacillus plantarum*, *Lactobacillus paracasei*, and *Lactobacillus bulgaricus* boosted NK cell activation and cytokine secretion [59]. NK cells expanded with probiotics and feeder cells displayed improved expansion, longer lifespan, increased cytotoxicity, and higher cytokine secretion compared to those expanded with feeder cells alone [50,57]. These expanded NK cells also showed a greater ability to induce pancreatic tumor killing and differentiation in both in vivo and in vitro studies [38,39]. They exhibited enhanced survival in the pancreatic tumor microenvironment (in vitro and mice model) due to higher anti-apoptotic protein levels like BCL2 and reduced pro-apoptotic proteins, allowing them to resist tumor-induced cell death. Elevated cytotoxic granules and Trail expression further improved their cytotoxic function [38,126]. Additionally, these expanded NK cells showed better regulatory functions, with most cells in an active cycling phase, increased expression of proliferation- and memory-associated genes, and enhanced activating receptors (e.g., CD16, CD56, Nkp30, Nkp44, Nkp46, NKG2D) while reducing inhibitory receptors like NKG2A, PD-1, and TIGIT [50,126]. Compared to NK cells expanded without probiotics, those expanded with probiotics demonstrated remarkable anti-cancer properties [50] (Figure 2).

The effectiveness of probiotics combined with feeder cell-expanded NK cell therapies has been shown in vivo using humanized mouse models, highlighting their potential for clinical use [38,39,57]. Infusing these expanded NK cells into healthy and pancreatic cancer-bearing humanized mice over eight weeks resulted in no toxicity, pain, distress, or adverse events such as cytokine-release syndrome (CRS) or immune effector cell-associated neurotoxicity syndrome (ICANS) [38]. Combining this NK cell-based therapy with chemotherapy or checkpoint inhibitors in pancreatic tumor-bearing humanized mice enhanced the effectiveness of both treatments [57]. Advances in NK cell expansion techniques have opened up exciting new therapeutic possibilities, and is under investigation for pancreatic cancer [53,132].

## 4. Exploring the Benefits of Adding Probiotics as a Supportive Therapy in Treating Pancreatic Cancer: Insights from Preclinical Studies

Several probiotics are being studied in preclinical settings for their potential benefits in pancreatic cancer. Probiotics can also boost immune responses and promote cancer cell death. The effects on pancreatic cancer depend on specific strains and the overall context, making strain selection crucial for achieving therapeutic goals (Table 1). In the mouse model, probiotics have been shown to enhance drug effectiveness, lessen chemotherapy side effects, and slow tumor progression [41]. *Lactobacillus paracasei* and *Lactobacillus reuteri* were found to enhance the efficacy of standard chemotherapy and improve the tolerance of chemotherapy in a transgenic mouse model of pancreatic cancer [41]. Oral administration of *Lactobacillus reuteri* and *Lactobacillus paracasei* in a pancreatic cancer mouse model reduced tumor load [68]. Ferrichrome from *Lactobacillus casei*, an iron chelate derivative, has demonstrated antitumor effects in refractory and 5-fluorouracil-resistant pancreatic cancer by regulating the tumor cell cycle through p53 activation [41,133]. Additionally, probiotics have been linked to increased tumor-infiltrating CD8+ T cells, higher IFN-γ expression, reduced inflammatory cytokines, and fewer postoperative complications like anastomotic leakage and bacteremia [38,39,134].

**Figure 3:**
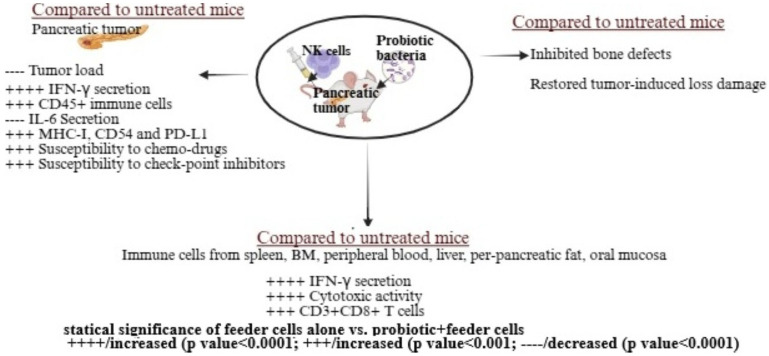
The combination of probiotics and NK cells improved immune function and reduced tumor load in a pancreatic tumor-bearing mouse model. These mice were orthotopically injected with human pancreatic tumors into the pancreas. One to two weeks after tumor implantation, they received NK cells via tail vein injection and were given 5 billion CFU probiotics orally every 48 h, starting one week before tumor implantation. At the end of the experiment, hu-BLT mice were sacrificed, and their tissues and tumors were collected and analyzed. Tumors from the probiotic and NK cell-treated group showed enhanced immune function, immune cell infiltration, and differentiation compared to the NK cell-only group. Tissues from the probiotic and NK cell-treated group had higher immune function and a greater percentage of CD3+CD8+ T cells compared to the NK cell-only group. Bones from the probiotic and NK cell-treated group displayed fewer defects and showed recovery from tumor-induced bone loss compared to the NK cell-only group. Illustration created with https://BioRender.com on 26 September 2025. https://app.biorender.com/illustrations/68cedfb1c9e8ad7e0f109b30?slideId=b6075359-d4a7-4a6b-8d8d-1b825340710f (accessed on 26 September 2025).

Oral administration of *Streptococcus thermophiles*, *Bifidobacterium longum*, *Bifidobacterium breve*, *Bifidobacterium infantis*, *Lactobacillus acidophilus*, *Lactobacillus plantarum*, and *Lactobacillus paracasei*, combined with NK cell therapy, significantly reduced tumor growth in pancreatic tumor-bearing humanized mice (Figure 3) [38,39]. Probiotic administration alone reduced the tumor load by approximately 1.3-fold, and combining probiotic feeding with NK cells reduced the tumor load by roughly 9-fold compared to untreated pancreatic tumor-bearing mice [38,39]. This combination enhanced NK cell activation in vivo, leading to tumors with increased PD-L1, CD54, and MHC-class I expression, slower growth, and greater sensitivity to checkpoint inhibitors and chemotherapy [39]. These changes likely improved CD8+ T cell-induced killing due to higher MHC-class I expression. Treated mice showed increased infiltration of human CD45+ immune cells, elevated IFN-γ secretion, and reduced IL-6 secretion in the tumor microenvironment, emphasizing the therapeutic benefits of probiotics and NK cells [39]. Probiotics also boosted tumor-infiltrating lymphocyte activity, particularly CD8+ T cell recruitment and cytotoxicity, enhancing cancer-specific immunity. Immune cells from peripheral blood, bone marrow, spleen, pancreas, oral mucosa, liver, and peri-pancreatic fatty tissue in probiotic and NK cell-treated mice exhibited higher cytotoxicity and cytokine secretion compared to those treated with NK cells alone [39]. Additionally, combining probiotics with NK cell therapy not only inhibited tumors but also reduced pancreatic tumor-induced bone defects and restored bone integrity (Figure 3) [38].

Preclinical research indicates that *Lactobacillus* strains might help reduce chemotherapy-induced inflammation and muscle wasting, potentially improving physical function, and indirectly impacting tumor progression through immunomodulation [135,136]. These findings suggest that probiotics could potentially enhance the effectiveness of standard therapy by reducing immunosuppression and inflammation in the tumor microenvironment. However, clinical validation is necessary to identify the best strains, dosage, duration, and combinations with therapy.

## 5. Clinical Studies on the Use of Probiotics in Human Pancreatic Cancer

While promising results have been observed in mice, the effects of probiotics on pancreatic cancer growth in humans are still under investigation. Initial studies emphasizing safety, feasibility, and mechanisms, while observational and metagenomic research provide valuable insights for future progress. Common strains like *Bifidobacteria* and *Lactobacillus* are often used in multistrain formulations, with optimal doses generally ranging from 10^8^ to over 10^10^ CFU daily [43,137]. Pancreatic cancer patient consuming *Streptococcus thermophiles*, *Bifidobacterium strains*, *Lactobacillus acidophilus*, *Lactobacillus plantarum*, *Lactobacillus paracasei*, and *Lactobacillus bulgaris* (125 billion CFU per capsule, three times daily for four weeks) demonstrated enhanced IFN-γ levels and increased cytotoxic activity in PBMCs and NK cells [138]. As of November 2025, clinical trials specifically exploring probiotics, microbiome modulation, or gut/tumor microbiota in pancreatic cancer remain limited but are steadily increasing [139]. No late-phase trials exclusively focus on probiotics for pancreatic cancer yet. Current trials prioritize supportive care and mitigating chemotherapy side effects rather than directly targeting tumor regression. Probiotic exposure, including strains like *Lactobacillus rhamnosus*, *Lactobacillus helveticus*, *Lactobacillus casei*, and *Saccharomyces boulardii*, has been associated with improved survival in pancreatic cancer patients under palliative care [140].

It has been found that *Clostridium butyricum* may play a role in improving the effectiveness of cancer treatments by supporting gut microbiome balance and boosting immune responses. Probiotics could not only help strengthen immunity but also ease some of the side effects associated with cancer therapies (NCT06998823). A recent study at Seoul National University Hospital looked into the effects of taking *Lactobacillus rhamnosus/Lactobacillus helveticus* 20 mg, *Lactobacillus casei/Lactobacillus rhamnosus* 250 mg, and *Saccharomyces boulardii* 250 mg on pancreatic cancer patients. This retrospective review focused on those undergoing palliative chemotherapy, aiming to assess overall survival (OS) and progression-free survival (PFS). Results showed that patients who took probiotics had significantly better overall survival than the control group, with median survival times producing a *p*-value of 0.026 between groups [68]. The LT-002 clinical trial (NCT06436976) is studying *Lactobacillus reuteri* ATG-F4 to assess its potential to enhance gut microbiome composition. It aims to alleviate chemotherapy-related side effects like cachexia, diarrhea, and inflammation, preserve physical performance and quality of life, and explore links between probiotic use and tumor progression, while tracking immune biomarkers. This study takes a mechanistic and translational approach, linking the microbiome, immune response, and chemotherapy tolerance in pancreatic cancer patients. It builds on earlier findings that certain Lactobacillus strains may impact tumor-associated macrophages via the AhR pathway.

A single-blind, randomized study (NCT06199752) looked into the combined immunomodulatory effects of synbiotics (probiotics and inulin prebiotics) and their impact on postoperative outcomes compared to probiotics alone. Ninety PDAC patients were divided into three groups: a placebo group, a probiotics group (receiving ten strains of *Lactobacillus*, *Bifidobacterium*, and *Streptococcus* at 25 billion CFUs), and a synbiotics group (probiotics plus inulin). The treatments began 14 days before surgery and continued for a month afterward. CD8+ T cell infiltration and IFN-γ expression were analyzed using immunohistochemistry, while levels of IL-1β, IL-6, and IL-10 were measured at various stages. Post-surgery, patients were monitored for short-term outcomes. Results showed a significant boost in CD8+ T cells and IFN-γ in the synbiotics group compared to the probiotics group. Inflammatory cytokines decreased more in the synbiotics group than in the others. Both probiotics and synbiotics reduced complications like anastomotic leakage, diarrhea, and abdominal distension, with the synbiotics group showing a notable reduction in bacteremia. These findings suggest that synbiotics may strengthen the immune system and minimize surgery-related issues [134]. Below is the list of the two most advanced clinical trials using probiotics in pancreatic cancer patients (Table 2).

Although research shows promising potential for probiotics in treating pancreatic cancer, some studies suggest certain gut bacteria might actually worsen tumor growth, highlighting the complex link between the microbiome and cancer [141]. Ongoing trials are key to better understanding these interactions, and more research is needed to draw clear conclusions about the safety and effectiveness of probiotics in this setting. Overall, current studies offer valuable insights into how probiotics could help manage pancreatic cancer, and as they advance, they aim to define their exact role in improving patient outcomes and integrating into existing treatments.

## 6. Challenges and Limitations in Using Probiotics for Clinical Application in Pancreatic Cancer in Humans

Probiotics used for clinical use for human pancreatic cancer face numerous biological, mechanistic, clinical, and manufacturing challenges. The majority of available data is generated using mouse models; however, mouse models do not always reflect human pancreatic immunobiology due to differences in microbiome composition, immune responses, AhR ligand-binding affinities, and metabolic pathways. Probiotics dosages and durations validated in preclinical studies are impractical for humans, due to human genetic diversity, microbiota differences, or diet-related interactions [142,143,144]. For safety and consistency, probiotic doses and formulations should be standardized, but this has not been carried out yet for pancreatic cancer patients [145,146,147]. Their use faces challenges such as strain-specific effects, individual variability, low survival rates, risks for people with weakened immune systems, formulation difficulties, limited clinical evidence, and regulatory concerns like antibiotic resistance [145,146,147]. Effective dosing depends on the strain and treatment goals, whether boosting immunity or slowing cell growth. Delivery methods like capsules or fermented foods differ in viability and bioavailability, and individual microbiomes can lead to mixed results, highlighting the need for personalized strategies.

Probiotics can interact with other treatments, with results varying by person and stage of cancer. Limited studies have yet to study the interaction of probiotics with chemotherapy, immunotherapies, or anti-inflammatory drugs to confirm whether this interaction is synergistic or antagonistic. In pancreatic cancer, probiotics may help by improving gut microbiota, triggering cancer cell death, and boosting immune responses, though effects depend on the strain, influencing apoptosis, immunity, and the tumor environment. Studies have shown that some strains, like *Bifidobacterium dentium*, could be harmful [141]. While probiotics can promote tumor cell death and modulate immunity, the exact molecular pathways and tumor-specific effects have not yet been explored fully [70]. Colonization and immune response vary depending on factors like dysbiosis, age, diet, genetics, gut health, and microbiota composition [145,146,147]. Differences in native flora, disease stage, prior antibiotic use, chemotherapy, and diet make responses unpredictable. In some cases, they enhance immune activity (stimulating Tregs or SCFA-driven anti-inflammatory pathways), but in others, they can suppress antitumor immunity (e.g., AhR activation in macrophages), making it hard to identify who might benefit [147,148]. In immunocompromised people, including those on chemotherapy, probiotics may cross a leaky gut barrier, rarely causing bacteremia or sepsis [147,148]. They may also face challenges reaching or colonizing pancreatic tissue due to physical barriers and the lack of a native pancreatic microbiome [149]. Long-term colonization depends on adhesion mechanisms like exopolysaccharides or biofilms, often supported by prebiotics. Surviving stomach acid, bile, and digestion is tough, but encapsulation methods like alginate or chitosan, tailored to the strain, can improve viability [145,146,147]. In the future, advances like strain engineering, nanoencapsulation, synbiotics, and personalized microbiome analysis could make probiotics more effective for cancer treatment and prevention.

To date, clinical findings on probiotics in pancreatic cancer are limited and often produce mixed results [140]. Some studies suggest they might help extend progression-free survival, while others warn that certain Lactobacillus strains could actually promote tumor growth by suppressing the immune system [140]. An abundance of *Bacteroides*, *Lactobacillus*, and *Peptoniphilus* in pancreatic tissues was found to be associated with decreased CD4+. CD8+ and CD45RO+ T cells and poor prognosis of pancreatic cancer patients [150]. Small sample sizes, retrospective designs, and varied patient groups make solid conclusions tricky. Current regulations and production standards do not quite match the therapeutic demands. While they hold promise as a preventive or supportive option, the evidence so far is not strong enough for regular use. Addressing these hurdles will require careful patient selection, well-structured trials, and more in-depth mechanistic studies. Larger, well-designed trials are needed to determine their safety and effectiveness. Exploring next-generation probiotics or engineered microbial blends that target tumor-promoting pathways looks promising. It is also important to study how probiotics interact with the tumor microenvironment microbiota, immune cells, and metabolites, and to consider postbiotics and paraprobiotics that might avoid colonization issues and reduce risks.

## 7. Role of Microbiome Stratification in Ensuring and Boosting the Therapeutic Benefits of Probiotics or Combined NK Cell Therapies

Probiotics could offer potential benefits as supportive treatments for pancreatic cancer, but their use needs to be personalized, carefully monitored, and supported by strong evidence. Important safety measures include selecting the right strains, watching dosages, pairing them with standard treatments, and evaluating each patient individually to prevent issues like immune suppression or unintended tumor growth. To ensure safety, especially for immunocompromised cancer patients, strict filtration systems are essential to remove contaminants before use. Cleanroom process control filters also play a key role in keeping environments sterile during therapy development and delivery. Probiotic-based pancreatic cancer therapies require multiple safeguards, such as accurate tumor targeting, controlled therapeutic release, immune system modulation, and close clinical monitoring to maximize benefits while reducing risks and maintaining microbiome health [151]. Engineered microbes should colonize only tumors, avoiding healthy tissues through methods like TME-responsive promoters or metabolic targeting. Cytotoxins like Theta toxin must activate only in tumor-specific conditions, with safety tools such as inducible promoters, kill switches, auxotrophy, or antibiotic sensitivity helping prevent uncontrolled growth and spread. Immune responses should be fine-tuned to enhance NK cell tumor-killing without excessive inflammation, while genetic stability must be preserved to avoid harmful gene transfer [152]. Careful dosing, timing, and biomarker tracking are vital for managing colonization and activity, and probiotic use should be balanced to protect beneficial microbes.

Microbiome stratification is about mapping an individual’s unique microbial makeup—whether in the gut, mouth, or even tumor tissue—to help guide personalized treatments [149]. Microbiome stratification—through genomic, metagenomic, or metabolomic analysis—helps identify patients who are more likely to respond well to specific probiotics or microbiome-focused treatments [153]. It also supports researchers in creating new therapies and predictive models for treatment success, while enabling pharmaceutical companies to design drug trials that account for microbiome health for better outcomes [154]. In pancreatic cancer, this approach can refine how probiotics, diet adjustments, and microbiota-based therapies are used by tapping into the complex interactions between microbes and the body [149,153]. Studies from both lab work and clinical trials suggest benefits like tailored prognostic insights, shaping the tumor’s environment, improving treatment outcomes, addressing therapy-resistant pathways, aiding early detection and biomarker-driven care, enabling targeted microbiome interventions, and minimizing potential risks.

The diversity of the tumor microbiome can influence survival outcomes, with higher intratumoral alpha-diversity and the presence of taxa like *Sphingomonas* and *Megasphaera* linked to longer survival, while elevated levels of *Bacteroides*, *Lactobacillus*, and *Peptoniphilus* are associated with poorer prognosis [140]. Profiling the microbiome can help identify high-risk patients for closer monitoring and tailored treatments. Certain probiotics, such as *Lactobacillus casei*, *Lactobacillus rhamnosus*, and *Saccharomyces boulardii*, may enhance CD8^+^ and CD4^+^ T-cell infiltration in the tumor environment, potentially boosting progression-free and overall survival in patients not eligible for curative surgery [140]. Targeted delivery can maximize immune benefits while minimizing harmful effects from bacteria like *Lactobacillus*, which can drive immunosuppression through AhR-mediated macrophage activity. The microbiome also affects how patients respond to chemotherapy and immunotherapy, with customized probiotics restoring butyrate-producing species and SCFAs to support cytostatic and anti-inflammatory effects. Stratification helps avoid strains that could promote tumor growth and informs probiotic strategies—whether through diet, antibiotics, or engineered consortia—to counteract immunosuppressive pathways from dysbiotic microbes [155].

Recent preclinical and translational studies have looked at how natural killer (NK) cells work alongside probiotic bacteria in pancreatic cancer models, focusing on eight strains, including *Lactobacillus* and *Bifidobacterium* species, and their impact on NK cell function. Research using humanized-BLT mouse models and ex vivo human NK cell tests has explored how long these effects can last. The durability of NK cells and probiotic-driven benefits after treatment depends on therapy type, microbiome recovery, and the lasting influence of probiotics. While immunotherapy and probiotics can spark noticeable but sometimes short-lived immune boosts, certain probiotic strains appear to influence NK cell activity more strongly [156]. In preclinical pancreatic cancer models, NK cells and probiotic combination have shown sustained boosts in NK cell cytotoxicity and tumor differentiation, lasting weeks and slowing tumor growth, with outcomes influenced by tumor stage, probiotic makeup, and immune context—though patient confirmation is still needed [38,39]. Keeping a balanced microbiome after treatment is key to supporting NK function and overall immune health. Probiotics may offer longer-term benefits if gut health is maintained, but results rely on a steady diet and consistent care. Without ongoing microbial support, NK cell levels can drop back to baseline soon after therapy ends. Benefits from NK cells and probiotic combos may fade if supplementation stops or dysbiosis occurs, and long-term monitoring is important to avoid harmful immune changes or cytokine imbalances [152]. Most findings are preclinical, and lasting effects in humans remain unproven.

In pancreatic cancer, breaking down the microbiome into specific groups allows for personalized and safe use of probiotics and other microbiome-based treatments [153]. Microbiome stratification helps doctors make the most of beneficial microbial interactions, improve immune monitoring, enhance treatment results, and possibly extend survival. Ongoing clinical trials, like PASS-01, which examine stool and tissue microbiota before and after chemotherapy, aim to refine these approaches and better integrate microbiome therapies into precision oncology. Pancreatic cancer patients those most likely to benefit from probiotics tend to have localized or borderline resectable tumors, lower-stage disease, or gut microbiomes well-suited to positive modulation (high Firmicutes, low Proteobacteria, and healthy diversity). In these cases, probiotics may boost progression-free and overall survival, but may offer little benefit—or even drawbacks—for advanced metastatic disease [149,157,158]. In short, microbiome-based stratification and targeted probiotic therapy show real promise as an add-on to standard pancreatic cancer treatments, particularly for early-stage patients with favorable microbiota and strong immune function. More studies are needed to establish clear guidelines and fully grasp the long-term advantages of microbiome stratification in cancer care plans.

## 8. Conclusions

This review highlights the significance of probiotics, particularly the strains listed in Table 1, in activating immune cells or signals to fight pancreatic cancer, their role in advancing cancer therapies, and enhancing the effectiveness of treatments. It emphasizes the benefits of probiotic bacteria and combination therapies, especially NK cell-based treatments, in reducing pancreatic tumor growth and spread. Studies performed in mouse models have shown that oral probiotics, alone or with NK cell infusions, help prevent tumor-related bone damage. Probiotics could help enhance the durability and antitumor activity of NK cells in pancreatic cancer by shaping the gut–tumor microbiota and influencing microbial metabolites, which can impact tumor infiltration and cytotoxic effects. However, in some situations, certain probiotics, particularly *Lactobacillus*, might suppress NK cell function indirectly through immunosuppressive pathways like AhR in macrophages. The overall effect largely depends on the specific context, highlighting the need for patient-tailored evaluation and further research. Challenges include limited clinical data available, strain-specific effects, and a lack of standardized clinical protocols (Table 3). Future research demands personalized microbiome profiling, optimized strains and dosages, and innovative delivery systems to develop safe and highly efficient treatments.

## Figures and Tables

**Table 2:** Clinical trials using probiotics in human pancreatic cancer.

*Probiotics*	Combined Therapy	Dose	Trial Phase	ClinicalTrials.gov Identifier	Patients Enrolled
*Clostridium butyricum *			NA	NCT06998823	120
*Lactobacillus reuteri * ATG-F4	oxaliplatin-based chemotherapy	4 × 10^10^ CFU daily for 12 weeks	Phase 2	NCT06436976	30
*Lactobacillus acidophilus*, *Bifidobacterium lactis*, *Lactobacillus plantarum*, *Lactobacillus paracasei*, *Bifidobacterium breve*, *Streptococcus thermophiles*, *Lactobacillus salivarius*, and *Bifidobacterium longum*	Surgical	Total: 2.5 × 10^10^ CFU	Phase 4	NCT06199752	90

**Table 3:** Strengths and limitations of probiotic use in pancreatic cancer.

Strengths	Limitations
Probiotics can impact a wide range of immune system functions linked to pancreatic cancer. Probiotics can affect innate immunity by influencing macrophages, dendritic cells, and natural killer (NK) cell activity, as well as adaptive immunity by shaping B-cell and T-cell responses, cytokine production, and the development of regulatory T cells (Tregs). Probiotics may also boost antigen presentation, adjust inflammatory signaling pathways, and support gut barrier health, all of which help maintain immune balance and could indirectly influence the progression of pancreatic tumors. Probiotics may boost the efficacy of NK cell-based and other conventional pancreatic cancer therapeutics. Probiotics may reduce tumor load, restore immune function, and restore bone defects in a mouse model of pancreatic cancer. Probiotics may increase the tolerance of chemotherapy in pancreatic cancer patients.	There is a lack of solid research and quality clinical trials, with most of the current findings coming from early lab work or small, mixed groups of human participants. It is still uncertain which strains work best, what the right dosage is, or how safe probiotics are for people with pancreatic cancer. Studies so far just do not provide enough proof to confirm benefits or set clear guidelines. Sometimes, taking probiotics could raise the toxicity associated with immunotherapy, perhaps due to their unpredictable effects on the body’s immune response.

## Data Availability

No new data were created or analyzed in this study. Data sharing is not applicable to this article.
